# Complimentary

**Published:** 1887-03

**Authors:** Jacob L. Williams


### COMPLIMENTARY.

Our readers will bear witness that this journal has not often indulged in self-glorification. We have received many letters of congratulation and praise that were warmly appreciated, but we feared that we should be liable to a charge of undue boasting if we printed them. The following, however, coming as it does from one of the oldest and most revered of our number, we cannot refrain from printing:

1 MT. VERNON ST., BOSTON, Feb. 4, 1887.

*Dear Mr. Editor:*—I am happy to enclose postal note for $2.50, for the continuance for 1887 of THE INDEPENDENT PRACTITIONER, which I consider the very best journal, in its line, that is published in the world.

I am much gratified with the solid and impregnable front it opposes to every attack on our department of practice. The article by Dr. Marvin, in the February number, shows an ability to comprehend and logically to elucidate fundamental scientific principles, that I do not believe can be exceeded in the ranks of the specialty or of the parent profession. Very truly yours,

JACOB L. WILLIAMS.

